# Supplementary material for: Prominent features of the amino acid mutation landscape in cancer
Source: PLoS One. 2017 Aug 24;12(8):e0183273. doi: 10.1371/journal.pone.0183273 (PMC5570307; doi:10.1371/journal.pone.0183273)
Supplement: S1 Fig — NMF identifies six mutation signatures from data aggregated by cancer subtype including signatures with (A) high R>H weights, (B) high A>T and R>H weights, (C) high E>K weights, (D) high E>K and E>Q weights, and complex signatures (E) and (F). Amino acid mutations to or from X represent mutations to or from stop codons. (G) The cophenetic distance and change in residual sum of squares for different choices for the number (k) of mutation signatures for the NMF analyses of data aggregated by cancer subtype. Solid lines represent NMF on the original data and dotted lines represent NMF on this data after randomization. Six to seven components offer a consistently good fit. (PDF) [file pone.0183273.s001.pdf]

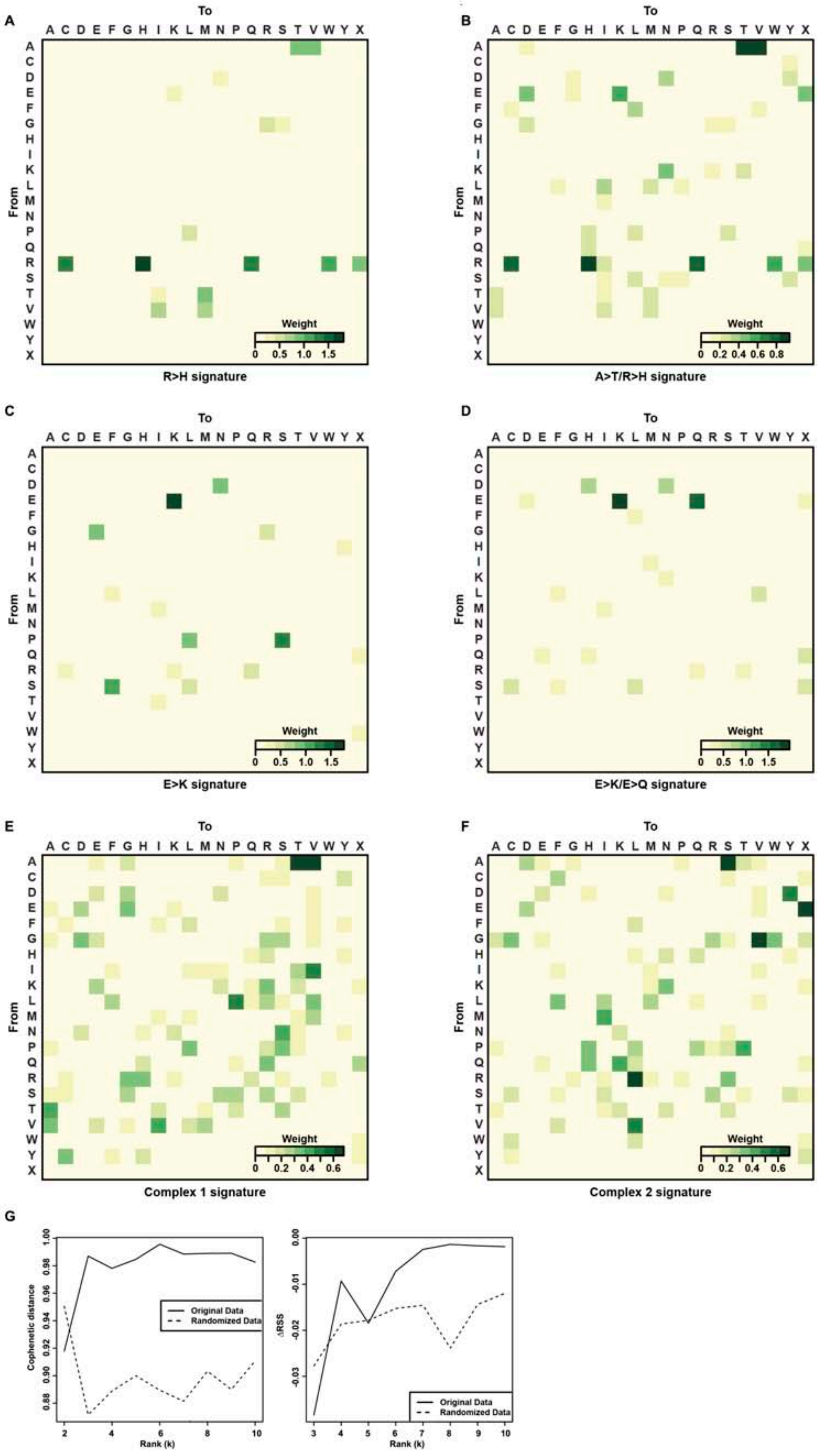

S1 Fig. Six nonsynonymous mutation signatures identified by NMF on aggregated data. NMF identifies six mutation signatures from data aggregated by cancer subtype including signatures with (A) high R>H weights, (B) high A>T and R>H weights, (C) high E>K weights, (D) high E>K and E>Q weights, and complex signatures (E) and (F). Amino acid mutations to or from X represent mutations to or from stop codons. (G) The cophenetic distance and change in residual sum of squares for different choices for the number (*k*) of mutation signatures for the NMF analyses of data aggregated by cancer subtype. Solid lines represent NMF on the original data and dotted lines represent NMF on this data after randomization. Six to seven components offer a consistently good fit.
