# Supplementary material for: Prominent features of the amino acid mutation landscape in cancer
Source: PLoS One. 2017 Aug 24;12(8):e0183273. doi: 10.1371/journal.pone.0183273 (PMC5570307; doi:10.1371/journal.pone.0183273)
Supplement: S2 Fig — (A) The first principal component separates cancers with many R>H mutations from those with many E>K mutations, and (B) the second principal component separates these cancers from those with more complex signatures. Amino acid mutations to or from X represent mutations to or from stop codons. (PDF) [file pone.0183273.s002.pdf]

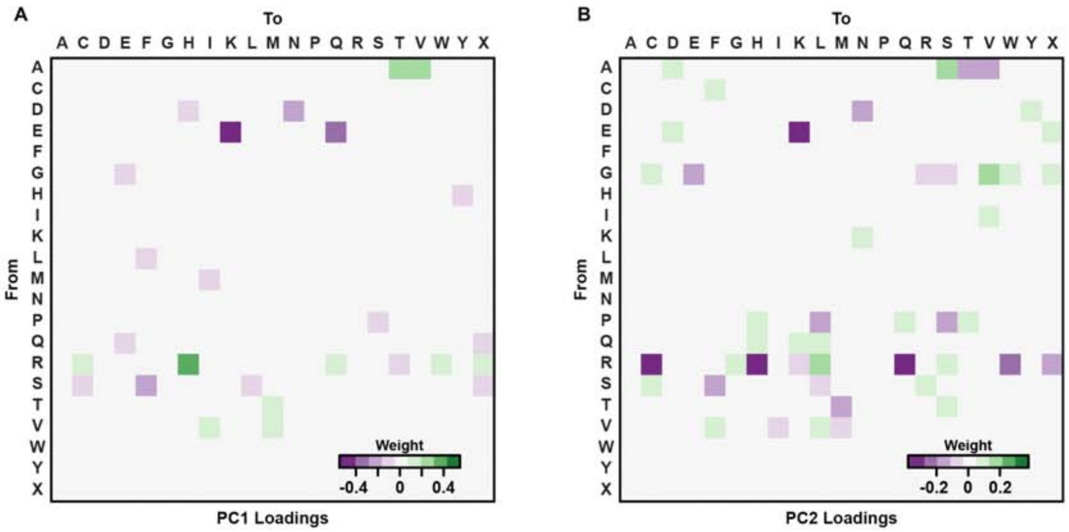

S2 Fig. Principal components analysis loadings on aggregated nonsynonymous mutations for the first two principal components.

(A) The first principal component separates cancers with many R>H mutations from those with many E>K mutations, and (B) the second principal component separates these cancers from those with more complex signatures. Amino acid mutations to or from X represent mutations to or from stop codons.
