# Supplementary material for: Prominent features of the amino acid mutation landscape in cancer
Source: PLoS One. 2017 Aug 24;12(8):e0183273. doi: 10.1371/journal.pone.0183273 (PMC5570307; doi:10.1371/journal.pone.0183273)
Supplement: S3 Fig — NMF identifies six mutation signatures from individual samples (only those with >10 total nonsynonymous mutations) including four signatures (A), (B), (C), (D) identified in the aggregated analysis and two other complex signatures, (E) and (F). Amino acid mutations to or from X represent mutations to or from stop codons. (G) The cophenetic distance and change in residual sum of squares for different choices for the number (k) of mutation signatures for the NMF analyses of data aggregated by individual samples. Solid lines represent NMF on the original data and dotted lines represent NMF on this data after randomization. Six to seven components offer a consistently good fit. (PDF) [file pone.0183273.s003.pdf]

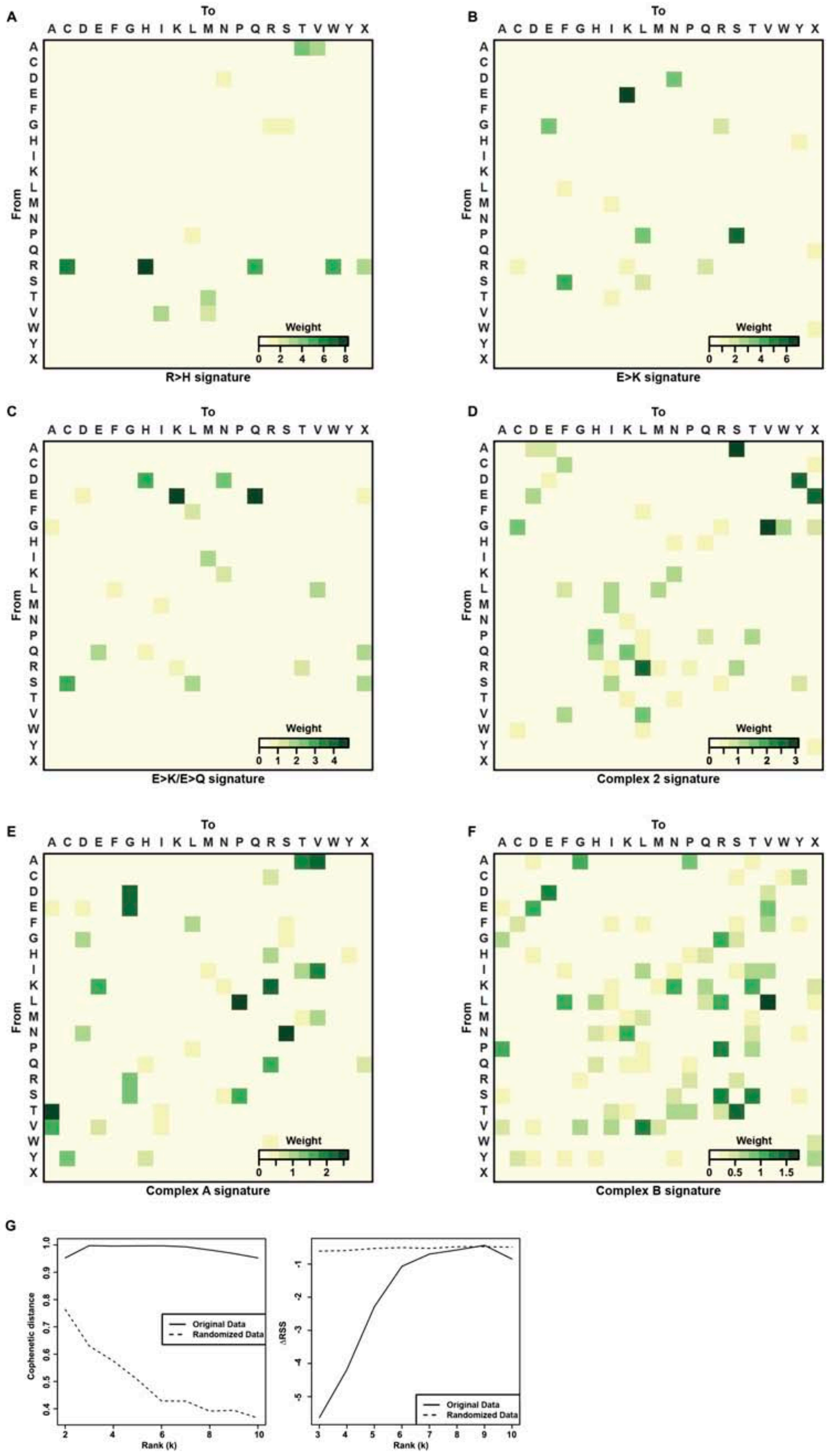

S3 Fig. Six nonsynonymous mutation signatures identified by NMF on individual samples.
NMF identifies six mutation signatures from individual samples (only those with >10 total nonsynonymous mutations) including four signatures (A), (B), (C), (D) identified in the aggregated analysis and two other complex signatures, (E) and (F). Amino acid mutations to or from X represent mutations to or from stop codons. (G) The cophenetic distance and change in residual sum of squares for different choices for the number (*k*) of mutation signatures for the NMF analyses of data aggregated by individual samples. Solid lines represent NMF on the original data and dotted lines represent NMF on this data after randomization. Six to seven components offer a consistently good fit.
