# Supplementary material for: Prominent features of the amino acid mutation landscape in cancer
Source: PLoS One. 2017 Aug 24;12(8):e0183273. doi: 10.1371/journal.pone.0183273 (PMC5570307; doi:10.1371/journal.pone.0183273)
Supplement: S4 Fig — Six mutation signatures identified by NMF from individual samples in the COSMIC database (only those with >10 total nonsynonymous mutations) used for validation of NMF results from Alexandrov data. Amino acid mutations to or from X represent mutations to or from stop codons. (A), (C), (E), and (F) match with previously found signatures from the Alexandrov data. (PDF) [file pone.0183273.s004.pdf]

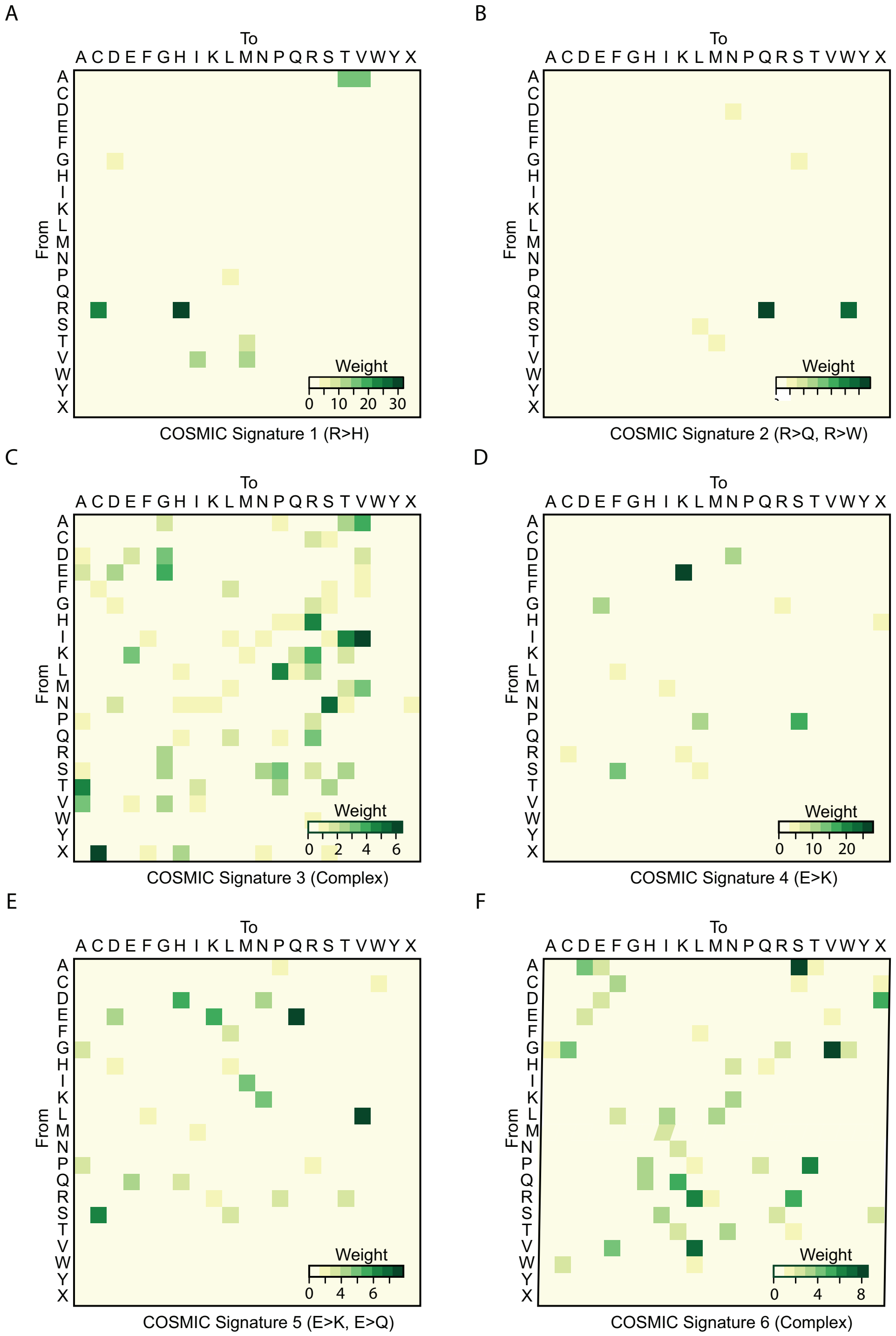


**S4 Fig. Six nonsynonymous mutation signatures identified by NMF on individual samples from the COSMIC database.** Six mutation signatures identified by NMF from individual samples in the COSMIC database (only those with >10 total nonsynonymous mutations) used for validation of NMF results from Alexandrov data. Amino acid mutations to or from X represent mutations to or from stop codons. (A), (C), (E), and (F) match with previously found signatures from the Alexandrov data.
