# Supplementary material for: Prominent features of the amino acid mutation landscape in cancer
Source: PLoS One. 2017 Aug 24;12(8):e0183273. doi: 10.1371/journal.pone.0183273 (PMC5570307; doi:10.1371/journal.pone.0183273)
Supplement: S1 Table — All Filters: entire filtering schema (Alexandrov et al., 2013) applied; No Filtering: none of the filters applied; Seq. Artifact Filter: only mutations defined as sequencing artifacts (Alexandrov et al., 2013) filtered. The expected range of mutation counts for the driver genes in cancers are based on reported mutation frequencies in the cancer genetics field and reported p53 mutation rates (see Methods). (PDF) [file pone.0183273.s005.pdf]

S1 Table. Expected and observed mutation frequency for cancer driver genes. All Filters: entire filtering schema (Alexandrov *et al.*, 2013) applied; No Filtering: none of the filters applied; Seq. Artifact Filter: only mutations defined as sequencing artifacts (Alexandrov *et al.*, 2013) filtered. The expected range of mutation counts for the driver genes in cancers are based on reported mutation frequencies in the cancer genetics field and reported p53 mutation rates (see Methods).

Gene	Cancer Type	# of samples	No filtering	All filters	Seq. artifact filter	Expected frequency
BRAF	Melanoma	396	193	21	193	158—198
IDH1	Low-Grade Glioma	217	165	0	149	163—184
IDH1	AML	154	14	0	4	12—18
IDH1	Glioblastoma	98	12	0	11	0—5
TP53	Colorectum	559	327	157	277	242
TP53	Head and Neck	380	200	101	180	162
TP53	Pancreas	113	27	14	25	39
TP53	Stomach	212	83	32	70	69
TP53	Liver	88	20	18	19	27
TP53	Breast	963	245	125	212	220
