# Supplementary material for: Prominent features of the amino acid mutation landscape in cancer
Source: PLoS One. 2017 Aug 24;12(8):e0183273. doi: 10.1371/journal.pone.0183273 (PMC5570307; doi:10.1371/journal.pone.0183273)
Supplement: S2 Table — Data was filtered using sequencing artifacts filter and any samples containing only synonymous mutations were eliminated from analysis (see Methods). Calculated is the mean number of nonsynonymous mutations per sample. Pilocytic astrocytoma samples were eliminated from analyses due to low frequency of nonsynonymous mutations. (PDF) [file pone.0183273.s006.pdf]

S2 Table. Total nonsynonymous mutation counts by cancer type. Data was filtered using sequencing artifacts filter and any samples containing only synonymous mutations were eliminated from analysis (see Methods). Calculated is the mean number of nonsynonymous mutations per sample. Pilocytic astrocytoma samples were eliminated from analyses due to low frequency of nonsynonymous mutations.

Cancer Type	Total # nonsyn. mutations	# of samples	Mutations per sample
ALL	1375	141	9.7518
AML	1503	151	9.9536
Bladder	28212	136	207.4412
Breast	36973	963	38.3936
CLL	1525	131	11.6412
Cervix	5729	38	150.7632
Colorectum	181751	559	325.1360
Esophageal	17740	146	121.5068
Glioblastoma	11336	96	118.0833
Low-Grade Glioma	17035	216	78.8657
Head and Neck	44525	380	117.1711
Kidney Chromophobe	1094	65	16.8308
Kidney Clear Cell	18936	325	58.2646
Kidney Papillary	4356	100	43.5600
Liver	4921	88	55.9205
Lung Adeno	156766	660	237.5242
Lung Small Cell	12631	70	180.4429
Lung Squamous	48343	176	274.6761
Lymphoma B-cell	1765	48	36.7708
Medulloblastoma	1328	97	13.6907
Melanoma	169273	396	427.4571
Myeloma	2479	69	35.9275
Neuroblastoma	3764	210	17.9238
Ovary	21032	471	44.6539
Pancreas	3974	113	35.1681
Pilocytic Astrocytoma	103	47	2.1915
Prostate	2411	330	7.3061
Stomach	63650	212	300.2358
Thyroid	4377	304	14.3980
Uterus	134100	240	558.7500
